# Protocol for a systematic review and individual participant data meta-analysis of optimizing oxygen therapy in critically ill patients

**DOI:** 10.3389/fmed.2024.1356557

**Published:** 2024-08-23

**Authors:** Xiaobo Yang, Yaqi Ouyang, Jiqian Xu, You Shang

**Affiliations:** Department of Critical Care Medicine, Union Hospital, Tongji Medical College, Huazhong University of Science and Technology, Wuhan, China

**Keywords:** oxygen therapy, intensive care unit, meta-analysis, individual participant data, systematic review, protocol

## Abstract

**Background:**

Oxygen therapy is a cornerstone treatment of critically ill patients in the intensive care unit (ICU). Whether lower oxygenation therapy brings superior survival outcomes to higher oxygenation therapy is unknown.

**Methods:**

We will search electronic databases: PubMed, Embase, Web of Science, the Cochrane Central Register of Controlled Trials (CENTRAL), International Clinical Trials Registry Platform (ICTRP), and ClinicalTrials.gov from inception to 1 January 2024. Two authors will independently screen for all eligible clinical studies. Emails will be sent for individual participant data. The statistical analyses will be conducted using STATA 15.0 software.

**Results:**

We will evaluate the efficacy of lower oxygenation therapy compared with higher oxygenation therapy based on individual participant data.

**Conclusion:**

This study will offer clinical evidence for oxygen therapy in ICU patients.

## 1 Introduction

Oxygen is commonly used in medical settings, especially for critically ill patients who may have an increased need for oxygen (1). However, it is important to note that too much oxygen, or hyperoxia, can actually be harmful to some patients (2). This is particularly true for those who have had a myocardial infarction (MI) or have been resuscitated from cardiac arrest. Recent studies have shown that hyperoxia can cause further damage to the heart in patients with ST-elevation MI who are not experiencing hypoxia (3). In addition, arterial hyperoxia after cardiac arrest may lead to higher rates of in-hospital mortality (4, 5). In a preplanned secondary analysis of targeted hypothermia vs. targeted normothermia after out-of-hospital cardiac arrest, Robba et al. (6) found that the time exposure of hyperoxemia was significantly associated with mortality. A systematic review and meta-analysis examining the association of hyperoxemia with survival and neurological outcomes included 10 observational studies of patients with refractory cardiogenic shock or refractory cardiac arrest treated with venoarterial extracorporeal membrane oxygenation. Tigano et al. (7) found that severe hyperoxemia may be associated with worse survival and neurological outcomes in these patients. As a result, medical professionals now recommend a peripheral oxygen saturation (SpO_2_) level of 94–98% for these patients (8).

Two systematic reviews and meta-analyses on trials of oxygen therapy have been performed (9, 10). However, recent studies, such as the ICU-ROX study, the PILOT study, the LOCO_2_ study, and the HOT-ICU study, were not included (11–14). To our knowledge, there has been no meta-analysis of individual participant data from trials of oxygen therapy in critically ill patients. In this study, we will evaluate the efficacy of low oxygen therapy in critically ill patients to provide evidence for oxygen therapy in the ICU.

## 2 Methods

### 2.1 Study registration

This meta-analysis was registered with PROSPERO on 30 September 2023 (registration number CRD42023464558). The Preferred Reporting Items for Systematic Review and Meta-Analysis Protocols (PRISMA-IPD) will be followed.

### 2.2 The inclusion criteria

#### 2.2.1 Types of studies

Only randomized controlled trials (RCTs) are considered for inclusion. Animal experiments, case reports, non-RCTs, secondary analysis of RCTs, and reviews will be excluded.

#### 2.2.2 Types of participants

Adults admitted into ICUs, including cardiovascular ICUs, neurological ICUs, surgical ICUs, medical ICUs, and general ICUs, are eligible for our study.

#### 2.2.3 Types of interventions

The interventions are lower oxygenation therapy and higher oxygenation therapy.

#### 2.2.4 Types of outcomes

The main outcome is 28-day mortality. The secondary outcomes are 60-day mortality and 90-day mortality.

### 2.3 Collection and analysis of data

#### 2.3.1 Search strategy

Author XY will carry out a thorough search in electronic databases: PubMed, Embase, Web of Science, the CENTRAL, ICTRP, and ClinicalTrials.gov from inception to 1 January 2024.

#### 2.3.2 Selection of studies

All authors will study PRISMA-IPD. Authors YO and JX will independently review the titles and abstracts of all retrieved studies for their eligibility, and the references for other possible eligible studies. All repetitions and studies that do not meet the enrollment criteria will be excluded. The included studies will then be cross-checked, and any uncertainties will be resolved by discussion with authors XY and YS. Emails will be sent by authors XY and YS to the corresponding authors to request data on gender, age, race, country, intervention group, the presence of mechanical ventilation at enrollment, the presence of shock and the type of shock at enrollment, the presence of MI at enrollment, SpO_2_, PaO_2_, time to death since enrollment, and the living status at 28th day, 60th day, and 90th day of each participant from all included studies. For those who do not respond, another five emails will be sent 1 week or 2 weeks after the previous email. Any ambiguous information will be cleared by discussion with the corresponding authors.

#### 2.3.3 Assessment of risk of bias and quality of evidence

**Figure 1:**
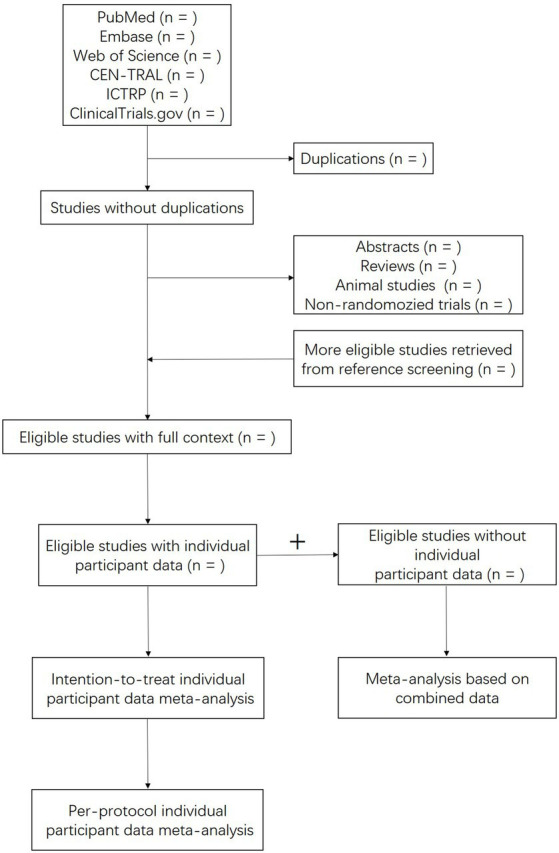
Flow diagram of studies selection and data synthesis.

The risk of bias and quality of evidence will be assessed using Cochrane’s “Risk of bias 2” tool. The following domains will be involved: sequence generation, allocation concealment, blinding, and completeness of outcomes and measures. The diagram of this study is shown in Figure 1.

### 2.4 Statistical analysis

#### 2.4.1 Synthesis of data

If there are two groups of higher oxygenation therapy, they will be combined as one higher oxygenation therapy group. Individual participant data will be combined according to the intention-to-treat group.

#### 2.4.2 Measures of effect

Author XY will perform the statistical analyses with Stata 15.0 and its command called IPDMETAN, which was designed for two-stage individual participant data meta-analyses of any measures of effect. The hazard ratio (HR) and a 95% confidence interval (CI) of mortality will be calculated.

#### 2.4.3 Assessment of heterogeneity

The heterogeneity will be assessed by Cochrane’s *Q*-test and *I*^2^ will be presented.

#### 2.4.4 Sensitivity analysis

Sensitivity analysis will be conducted in two ways. First, aggregated data from studies without individual participant data will be included to perform a sensitivity analysis. Second, per-protocol individual participant data will be combined to conduct a meta-analysis. Per-protocol is defined as the oxygenation target within the predefined interval of each group from each included study.

#### 2.4.5 Assessment of reporting bias

A funnel plot and the Egger test will be used to assess reporting bias.

#### 2.4.6 Subgroup analysis

Subgroup analyses will be conducted as per primary analysis if sufficient data are available: age ≥65 years vs. age <65 years, invasive mechanically ventilated (IMV) vs. non-IMV, shock vs. non-shock, acute myocardial infarction (AMI) vs. non-AMI, stroke vs. non-stroke, and cardiac arrest vs. non-cardiac arrest at enrollment.

## 3 Discussion

Oxygen therapy has been used, and the toxicity of supranormal oxygen has been recognized for more than a century (15). Providing the appropriate amount of oxygen is a balance of potential benefits and risks. For some critically ill patients, oxygen is a life-or-death therapy, and both hypoxia and hyperoxia are associated with an increased risk of death (16). The benefits and risks of appropriate oxygen therapy are most likely to be established in ICU patients because of the convenience of targeted oxygen therapy and the relatively higher mortality compared with patients in general wards. In recent years, several large randomized trials comparing low and high oxygen therapy in critically ill patients have been reported, including the ICU-ROX trial, the PILOT trial, the LOCO_2_ trial, and the HOT-ICU trial (11–14). A meta-analysis including these trials is needed.

Compared with meta-analysis of aggregated data, meta-analysis of individual participant data is more powerful (17). Another strength of this meta-analysis will be the two ways of sensitivity analysis. It is not easy to obtain individual participant data. Meta-analysis of combinations of individual participant data from some trials and aggregated data from other trials is an important supplement to meta-analysis of individual participant data. In the higher oxygenation group, the oxygenation target is easily achieved. However, in the lower oxygenation group, the actual oxygenation index is usually higher than the predefined upper limit of the target. For example, the predefined upper limit of the SpO_2_ target was 92% in Panwar’s et al. (18) study and 90% in the PILOT study (12); the actual SpO_2_ was 93.4 and 94%, respectively, in the two studies.

The different kinds of patients are a challenge for oxygen therapy and this meta-analysis. We aim to include all types of patients admitted to different types of ICUs. The overall effect of oxygen therapy on mortality will be examined. Subgroup analyses will be performed, and the results may serve as hints for further randomized trials.

## Author contributions

XY: Conceptualization, Data curation, Formal analysis, Funding acquisition, Investigation, Methodology, Software, Writing – original draft. YO: Data curation, Investigation, Writing – review & editing. JX: Investigation, Writing – review & editing. YS: Investigation, Supervision, Writing – review & editing.

## References

[1] Martin DS Grocott MPW. Oxygen therapy in critical illness: precise control of arterial oxygenation and permissive hypoxemia. Crit Care Med. (2013) 41:423–32. doi: 10.1097/CCM.0b013e31826a44f6, PMID: 23263574

[2] Budinger GRS Mutlu GM. Balancing the risks and benefits of oxygen therapy in critically III adults. Chest. (2013) 143:1151–62. doi: 10.1378/chest.12-1215, PMID: 23546490 PMC3616683

[3] Stub D Smith K Bernard S Nehme Z Stephenson M Bray JE . Air versus oxygen in ST-segment-elevation myocardial infarction. Circulation. (2015) 131:2143–50. doi: 10.1161/CIRCULATIONAHA.114.014494, PMID: 26002889

[4] Kilgannon JH Jones AE Parrillo JE Dellinger RP Milcarek B Hunter K . Relationship between supranormal oxygen tension and outcome after resuscitation from cardiac arrest. Circulation. (2011) 123:2717–22. doi: 10.1161/CIRCULATIONAHA.110.001016, PMID: 21606393

[5] La Via L Astuto M Bignami EG Busalacchi D Dezio V Girardis M . The effects of exposure to severe hyperoxemia on neurological outcome and mortality after cardiac arrest. Minerva Anestesiol. (2022) 88:853–63. doi: 10.23736/S0375-9393.22.16449-7, PMID: 35319851

[6] Robba C Badenes R Battaglini D Ball L Sanfilippo F Brunetti I . Oxygen targets and 6-month outcome after out of hospital cardiac arrest: a pre-planned sub-analysis of the targeted hypothermia versus targeted normothermia after Out-of-Hospital Cardiac Arrest (TTM2) trial. Crit Care. (2022) 26:323. doi: 10.1186/s13054-022-04186-8 36271410 PMC9585831

[7] Tigano S Caruso A Liotta C LaVia L Vargas M Romagnoli S . Exposure to severe hyperoxemia worsens survival and neurological outcome in patients supported by veno-arterial extracorporeal membrane oxygenation: a meta-analysis. Resuscitation. (2024) 194:110071. doi: 10.1016/j.resuscitation.2023.110071, PMID: 38061577

[8] Olasveengen TM De Caen AR Mancini ME MacOnochie IK Aickin R Atkins DL . 2017 international consensus on cardiopulmonary resuscitation and emergency cardiovascular care science with treatment recommendations summary. Circulation. (2017) 136:e424–40. doi: 10.1161/CIR.0000000000000541, PMID: 29114010

[9] Chu DK Kim LH-Y Young PJ Zamiri N Almenawer SA Jaeschke R . Mortality and morbidity in acutely ill adults treated with liberal versus conservative oxygen therapy (IOTA): a systematic review and meta-analysis. Lancet. (2018) 391:1693–705. doi: 10.1016/S0140-6736(18)30479-3, PMID: 29726345

[10] van der Wal LI Grim CCA van Westerloo DJ Schultz MJ de Jonge E Helmerhorst HJF. Higher versus lower oxygenation strategies in the general intensive care unit population: a systematic review, meta-analysis and meta-regression of randomized controlled trials. J Crit Care. (2022) 72:154151. doi: 10.1016/j.jcrc.2022.154151, PMID: 36182731

[11] Mackle D Bellomo R Bailey M Beasley R Deane A Eastwood G . Conservative oxygen therapy during mechanical ventilation in the ICU. N Engl J Med. (2020) 382:989–98. doi: 10.1056/NEJMoa1903297 31613432

[12] Semler MW Casey JD Lloyd BD Hastings PG Hays MA Stollings JL . Oxygen-saturation targets for critically ill adults receiving mechanical ventilation. N Engl J Med. (2022) 387:1759–69. doi: 10.1056/NEJMoa2208415, PMID: 36278971 PMC9724830

[13] Barrot L Asfar P Mauny F Winiszewski H Montini F Badie J . Liberal or conservative oxygen therapy for acute respiratory distress syndrome. N Engl J Med. (2020) 382:999–1008. doi: 10.1056/NEJMoa1916431, PMID: 32160661

[14] Schjørring OL Klitgaard TL Perner A Wetterslev J Lange T Siegemund M . Lower or higher oxygenation targets for acute hypoxemic respiratory failure. N Engl J Med. (2021) 384:1301–11. doi: 10.1056/NEJMoa2032510, PMID: 33471452

[15] Singer M Young PJ Laffey JG Asfar P Taccone FS Skrifvars MB . Dangers of hyperoxia. Crit Care. (2021) 25:440. doi: 10.1186/s13054-021-03815-y 34924022 PMC8686263

[16] Hochberg CH Semler MW Brower RG. Oxygen toxicity in critically ill adults. Am J Respir Crit Care Med. (2021) 204:632–41. doi: 10.1164/rccm.202102-0417CI, PMID: 34086536 PMC8521700

[17] Riley RD Lambert PC Abo-Zaid G. Meta-analysis of individual participant data: rationale, conduct, and reporting. BMJ. (2010) 340:c221. doi: 10.1136/bmj.c221, PMID: 20139215

[18] Panwar R Hardie M Bellomo R Barrot L Eastwood GM Young PJ . Conservative versus liberal oxygenation targets for mechanically ventilated patients: a pilot multicentre randomized controlled trial. Am J Respir Crit Care Med. (2016) 193:43–51. doi: 10.1164/rccm.201505-1019OC, PMID: 26334785

